# Shear bond strength of artificial teeth used with CAD/CAM PMMA versus heat cure acrylic resins for complete denture - A systematic review

**DOI:** 10.6026/9732063002001095

**Published:** 2024-09-30

**Authors:** Rubina Tabassum, Gaurang Mistry, Jinal Prajapati, Ashwini Kini, Amit Pokharkar, Sanpreet Singh Sachdev

**Affiliations:** 1 Department of Prosthodontics, D.Y. Patil Deemed to be University, School of Dentistry, Navi, Mumbai, Maharashtra, India; 2 Department of Oral Pathology and Microbiology, Bharati Vidyapeeth (Deemed to be University) Dental College and Hospital, Navi, Mumbai, Maharashtra, India

**Keywords:** Complete Dentures, Heat-cure resins, Shear Bond Strength, PMMA

## Abstract

The present systematic review evaluates the shear bond strength of artificial teeth bonded to CAD/CAM PMMA and heat-cure acrylic resins used in complete denture manufacturing. The study aims to determine the superior bonding material, contributing to enhanced denture durability and patient satisfaction. A comprehensive search of databases including PubMed, MEDLINE, DOAJ, Cochrane Library and Scopus was conducted, adhering to PRISMA 2020 guidelines. Studies were assessed for inclusion based on specific criteria, and the data were analyzed using Review Manager (RevMan) 5.3. The findings suggest that while both materials provide adequate bond strength, differences exist that may influence material choice in clinical practice.

## Background:

Complete dentures play a vital role in restoring the functionality and aesthetics of edentulous patients. The durability and performance of these prostheses depend significantly on the strength of the bond between the artificial teeth and the denture base material [1]. Traditionally, heat-cure acrylic resins have been the material of choice due to their ease of manipulation, cost-effectiveness, and satisfactory mechanical properties [2]. However, the advent of CAD/CAM (Computer-Aided Design/Computer-Aided Manufacturing) technology has introduced new materials like CAD/CAM PMMA (Polymethylmethacrylate) that promise superior consistency and performance due to their controlled manufacturing processes [3]. Despite the widespread adoption of CAD/CAM technology in various aspects of prosthodontics, its application in denture-based fabrication is still evolving [4]. The bond strength between artificial teeth and CAD/CAM PMMA bases is of particular interest as it directly impacts the prosthesis' longevity and patient satisfaction [5]. The exact influence of this advanced material on the adhesive strength when compared to conventional heat-cure acrylic resins is still under investigation, and existing studies show mixed results [6,7]. This systematic review aims to critically evaluate and compare the shear bond strength of artificial teeth bonded to CAD/CAM PMMA bases against those bonded to heat-cure acrylic resin bases. By synthesizing current evidence, this review seeks to determine if CAD/CAM PMMA provides a significant advantage over traditional materials, thereby guiding clinical decisions in denture fabrication.

## Methods:

## Search strategy:

To gather relevant studies, a comprehensive search was conducted across multiple electronic databases, including PubMed, Scopus, Web of Science, and Cochrane Library. The search strategies combined terms related to "CAD/CAM PMMA", "heat-cure acrylic resin", "artificial teeth", "complete dentures" and "shear bond strength". The search was restricted to studies published in English between January 2000 and December 2023.

## Inclusion and exclusion criteria:

Studies were included in the review if they: (1) compared the shear bond strength between artificial teeth and CAD/CAM PMMA versus heat-cure acrylic resins, (2) used standardized testing methods for SBS evaluation and (3) were peer-reviewed. Exclusion criteria included studies that did not specifically compare the two materials, case reports, reviews, and those with incomplete data.

## Data extraction and quality assessment:

Two independent reviewers screened the titles and abstracts of the identified studies. Full-text articles were then retrieved and assessed for eligibility. Data extraction was performed using a standardized form, capturing details on study design, materials used, sample size, methods of bond strength testing, and outcomes. The quality of the included studies was assessed using the Cochrane risk-of-bias tool for randomized studies or appropriate quality assessment tools for non-randomized studies.

## Data synthesis and analysis:

A qualitative synthesis of the findings was conducted due to the heterogeneity of the study designs, materials and testing methods. Where feasible, a meta-analysis was performed using a random-effects model to account for variations among studies. The results were presented as mean differences with 95% confidence intervals.

## Results:

## Study characteristics:

Eight studies were included in this systematic review whose general characteristics are mentioned in Table 1 [8, 9, 10, 11, 12, 13, 14-15]. All the studies were conducted *in vitro*. These studies were conducted in different parts of the world, New Zealand, Egypt, Korea, Croatia, Thailand, Iran, and Greece. The intervention group was CAD/CAM-manufactured denture bases while control was conventional heat-cured resin denture bases. The commonly used CAD/CAM was Ivoclar Vivodent, while the heat cure resin used was Vertex Rapid. The conclusions of all studies indicated that teeth bonded to heat-polymerized resins produced the highest bond strength as compared to CAD/CAM denture bases.

## Quality assessment of included studies:

All the included studies showed a low risk of bias except Helal 2022, which showed a Moderate risk of bias. In a study by Helal 2022, information related to sample preparation and operator was not adequately mentioned, contributing to moderate risk in this study (Table 2).

## Meta-analysis:

Data synthesis was carried out using a descriptive synthesis, with a summary of the characteristics of each included study. For quantitative synthesis, a summary of the combined estimate related to the intervention effect was calculated as a mean of the differences in the effects of post-intervention in individual studies.

## Effect measures:

Effect measures refer to statistical constructs that compare outcome data between two intervention groups. The standardized mean difference is used as a summary statistic in a meta-analysis when the studies all assess the same outcome but measure it in a variety of ways. In this circumstance, it is necessary to standardize the results of the studies to a uniform scale before they can be combined. Hence for quantitative assessment in this study, standardized mean difference (SMD) was used as an effect measure. Meta-analysis was conducted on studies providing data on similar outcomes.

## Bond strength according to different types of denture teeth used:

Four studies used acrylic teeth with CAD/CAM and heat-cured denture bases. The pooled bond strength obtained was 0.03[-3.60, 3.66] indicating that the bond strength of acrylic teeth was greater with CAD/CAM denture base as compared to heat-cured denture base. Overall, the results were not statistically significant (p>0.05), with high heterogeneity (I2=98%). Five studies used composite teeth with CAD/CAM and heat-cured denture bases. The pooled bond strength obtained was 0.74[-1.58, 3.07] indicating that the bond strength of composite teeth was greater with CAD/CAM denture base as compared to heat-cured denture base. Overall, the results were not statistically significant (p>0.05), with high heterogeneity (I2=97%). Three studies used cross-linked teeth with CAD/CAM and heat-cured denture bases. The pooled bond strength obtained was -1.33[-3.87, 1.21] indicating that the bond strength of cross-linked teeth was greater with a heat-cured denture base as compared to a CAD/CAM denture base. Overall, the results were not statistically significant (p>0.05), with high heterogeneity (I2=96%).

## Bond strength irrespective of the type of denture teeth used:

Three studies were included. The pooled bond strength obtained was -2.11[-4.88, 0.67] indicating that the bond strength was greater with a heat-cured denture base as compared to a CAD/CAM denture base. Overall, the results were not statistically significant (p>0.05), with high heterogeneity (I2=92%).

## Discussion:

The present systematic review aimed to compare the SBS of artificial teeth used with CAD/CAM PMMA versus heat-cure acrylic resins in complete denture manufacturing. The primary objective was to evaluate whether the advent of CAD/CAM technology has led to a significant improvement in the bonding characteristics of denture base materials compared to traditional heat-cure methods.

The bonding mechanism between artificial teeth and denture base resins is multifactorial, involving chemical, mechanical and physical interactions. CAD/CAM PMMA materials are synthesized under controlled conditions, ensuring uniform polymerization and minimal residual monomer content, which is hypothesized to contribute to superior bond strength [16]. On the other hand, heat-cure acrylic resins, while still widely used, may suffer from variations in polymerization depending on the processing technique, potentially leading to weaker bonds [17]. Surface treatment of artificial teeth plays a crucial role in enhancing SBS. Studies have indicated that the application of bonding agents or surface roughening techniques can significantly improve the bond strength of both CAD/CAM PMMA and heat-cure acrylic resins [7, 18]. However, the extent of this improvement may vary depending on the material properties and the compatibility between the artificial teeth and the denture base resin [19]. The findings of this review have significant clinical implications. Dentures with higher SBS are less likely to experience tooth detachment during function, leading to improved patient satisfaction and prosthesis longevity [20]. The results suggest that CAD/CAM PMMA may offer superior bonding properties when composite and acrylic teeth are used, which could translate to better clinical outcomes. On the other hand, for cross-linked teeth or when not considering the type of teeth used, heat-cured denture bases were found to be superior. However, it is essential to consider that the choice of material should also take into account factors such as cost, availability, and the specific needs of the patient [21]. This review has several limitations that must be acknowledged. The included studies were heterogeneous in terms of the methodologies used to assess SBS, including variations in sample preparation, testing protocols and the type of artificial teeth and denture base materials used. Additionally, the long-term clinical performance of these materials was not evaluated, limiting the ability to draw definitive conclusions about their durability in vivo. Future studies should aim to standardize testing methods and include long-term clinical evaluations to provide more comprehensive data. Future research should focus on developing standardized testing protocols for SBS evaluation, incorporating a larger sample size and exploring the impact of different surface treatments and bonding agents. Moreover, long-term clinical studies are necessary to validate the *in vitro* findings and to assess the durability of CAD/CAM PMMA versus heat-cure acrylic resin dentures in a clinical setting.

## Conclusion:

The present systematic review suggests that heat cure acrylic resins may exhibit superior shear bond strength compared to CAD/CAM PMMA when used in complete denture manufacturing. The bonding characteristics of heat cure acrylic resins can potentially lead to improved clinical outcomes and increased patient satisfaction. However, further research with standardized methodologies and long-term clinical evaluations is needed to confirm these findings and to establish definitive guidelines for the selection of denture base materials.

## Figures and Tables

**Figure 1:**
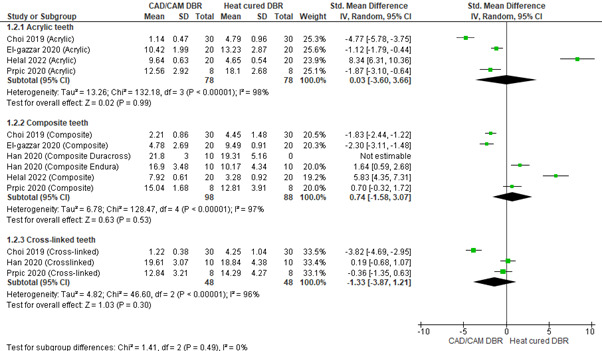
Pooled values for bond strength depending on the type of teeth used

**Figure 2:**
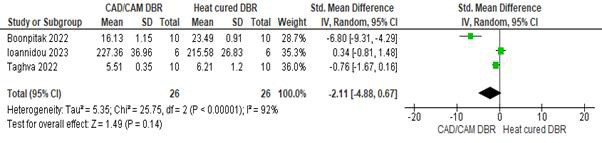
Pooled values for bond strength irrespective of the type of teeth

**Table 1:** Characteristics of included studies

**Study ID**	**Place of Study**	**Sample Size**	**Intervention**	**Control**	**Assessment of Bond Strength**	**Denture Teeth Used**	**Author Conclusions**
Choi 2019	New Zealand	30 per group	CAD/CAM (Ivoclar Vivodent, Liechtenstein)	Heat cured (Vertex dental)	N/A	1. Ivoclar SPE 2. DCL 3. MD (nanofillers)	-Highest fracture toughness with heat-cured DBRs. - Aging reduces bond strength. - CAD/CAM shows lower bond strength.
El-gazzar 2020	Egypt	20 per group	CAD/CAM denture base (Dr. Mat Dental CAD/CAM White Scan Spray, Istanbul, Turkey)	Heat-cured acrylic resins	Universal Testing Machine (Instron Corp, Canton, MA. USA).	1. Acrylic 2. Composite	-Non-significant lower tensile bond strength with CAD/CAM. - Higher bond strength with acrylic teeth.
Han 2020	Korea	10 each group	Pre-polymerized PMMA denture resin CAD/CAM disks 1. PMMA Block-pink 2. Vipi Block-Pink	Heat-polymerizing PMMA denture resin	Universal Testing Machine (OUT 05D, Oriental TM Corp., Gyeonggi-do, Korea)	1. Composite (Endura) 2. Composite (Duracross) 3. Cross-linked	- Comparable bond strengths between CAD/CAM and conventional methods.
Prpic 2020	Croatia	8 per group	CAD/CAM (milled) denture base resin (IvoBase CAD, Ivoclar Vivadent, Schaan, Liechtenstein)	Heat-polymerized acrylics	Universal shear bond strength testing machine (model LRX, Lloyd Instruments, Fareham, Great Britain) at 1 mm/min.	1. Acrylic 2. Composite 3. Cross-linked	- Similar bond strength values between CAD/CAM and heat-polymerized resins.
Boonpitak 2022	Thailand	10 each group	Surface-treated 3D-printed artificial teeth bonded with denture base resins, post-cured with heat	Surface-treated 3D-printed artificial teeth bonded to denture bases, heat-cured at 100°C for 30 min in a water bath	Universal Testing Machine (Shimadzu AGS-X, Kyoto, Japan)	N/A	- Greatest bond strength with heat-polymerized 3D-printed artificial teeth and DBRs.
Helal 2022	Egypt	20 per group	CAD/CAM	Heat-polymerized acrylic resin (Acrostone, Cairo, Egypt)	Universal Testing Machine (Instron Corp, Canton, MA. USA)	1. Acrylic 2. Composite	- Higher bond strength with CAD/CAM DBR.
Taghva 2022	Iran	10 each group	CAD/CAM (Vita Vionic, Germany)	Heat-cured acrylic resins (ProBase Hot, Ivoclar Vivadent)	Universal Testing Machine (ZwickRoell Zo20, Zwick, U1m, Germany) at 1 mm/min.	N/A	- Higher bond strength with heat-cured resin.
Ioannidou 2023	Greece	6 per group	PMMA CAD/CAM disc (PoliDent CAD/CAM disc, Volcja Draga, Slovenia)	Conventional heat curing method	Electromechanical loading frame (MTS Insight)	N/A	- CAD/CAM method can replace conventional methods in clinical practice.

**Table 2:** Quality assessment of included studies

**Study ID**	**Sample size**	**Random**	**Sample preparation**	**Operator**	**Measuring procedures**	**Statistical analysis**	**Total**	**Risk of bias**
Choi 2019	1	2	0	1	0	0	4	Low
El-gazzar 2020	1	2	0	0	0	0	3	Low
Han 2020	1	2	0	1	0	0	4	Low
Prpic 2020	1	2	0	0	0	0	3	Low
Boonpitak 2022	1	2	1	0	0	0	4	Low
Helal 2022	1	2	1	1	0	0	5	Moderate
Taghva 2022	1	2	0	1	0	0	4	Low
Ioannidou 2023	1	2	1	0	0	0	4	Low
